# Evaluating a Regression-Based Approach to Norming the Test of Masticating and Swallowing Solids (ToMaSS): A Comparison with Traditional Stratified Methods

**DOI:** 10.1007/s00455-026-10949-0

**Published:** 2026-05-08

**Authors:** Esther Guiu Hernandez, Nicole Lewis, Sarah Perry, Maggie-Lee Huckabee

**Affiliations:** 1 University of Canterbury Rose Centre for Stroke Recovery at Research at St. George’s Medical Centre, Christchurch, 8014 Canterbury New Zealand; 2 Department of Medicine, University of Otago Christchurch, Christchurch, New Zealand; 3 New Zealand Brain Research Institute, Christchurch, New Zealand

**Keywords:** ToMaSS, Mastication, Deglutition

## Abstract

The Test of Masticating and Swallowing Solids (ToMaSS) is a commonly-used adjunctive assessment to quantify solid bolus ingestion. However, published norms are characterised by relatively small sample sizes and wide age brackets, increasing the risk of skew, influence by outliers, and reduced statistical power. Regression-based approaches to norming may mitigate some of these limitations. To evaluate whether a regression-based approach offered a significant advantage over the traditional stratified approach for the refinement of ToMaSS norms. Prospective-retrospective design, with prospectively collected data combined with historical data from published and unpublished laboratory datasets. All data consisted of healthy adults (aged 20 to 80 + years) recruited from the general public. McFadden’s Pseudo R² and Root Mean Squared Error (MSE) were calculated and compared for both statistical approaches. Regression-based norms (GAM Gamma and COM-Poisson models) showed a modest improvement in model fit over traditional stratified norms, with McFadden’s Pseudo R² increasing by 6% to 43% across outcomes. MSE values followed a similar trend, with regression-based norms reducing prediction error by up to 12%. Findings support that a regression-based approach offered a significant advantage over a traditional stratified approach to constructing ToMaSS norms. Advantages included smaller sample size requirements and individualised estimates across continuous age ranges. This flexibility allows for a more nuanced understanding of individual ToMaSS performance, which may be of particular relevance to clinical populations where changes in the oral phase of swallowing may vary greatly within a short time frame.

## Introduction

Dysphagia (difficulty swallowing) is a common consequence of various medical disorders. Early detection of dysphagia is crucial to decrease the risk of medical morbidities and improve patient well-being. The clinical swallowing evaluation (CSE) is the initial step in screening a patient’s functional swallowing and to identify the risk of dysphagia [1]. However, this comprehensive evaluation relies predominantly on subjective identification of clinical signs and symptoms of dysphagia through observation [1, 2]. Previous research has documented that speech-language therapists varied in the items of the CSE they used, were inconsistent in performing the assessment, and varied in their clinical decision-making [1, 3–5]. Furthermore, McCullough et al. [6] found that less than 50% of CSE items used by clinicians had adequate inter- and intra-rater reliability. There is a need for quantitative assessment tools that reduce subjectivity of the CSE and allow for comparison to normative data.

Water-swallowing tests are among the most commonly-used clinical screening approaches. For example, the Timed-Water Swallowing Test (TWST) was designed as a quantitative assessment tool to assess liquid swallowing efficiency [7, 8]. Administration of the TWST requires individuals to drink 100–150 ml of water from a cup “as quickly as is comfortably possible” [8]. Three swallowing indices are recorded: the average volume per swallow (ml/swallow), average time per swallow (s/swallow), and a measure of efficiency, termed swallowing capacity (ml/s). Normative data have been collected in healthy adults with no swallowing difficulties using small sample sizes within each sex and age-stratified group [8–11]. The original publication provided limited clinical reference data for people with amyotrophic lateral sclerosis [8], and disease-specific data have further been published for individuals with Parkinsonism [12], head and neck cancer [13], idiopathic pulmonary fibrosis [14], stroke [15], and Huntington’s disease [16]. However, the TWST only assesses liquid ingestion and may not be suitable for patients who are unable to safely ingest liquid [8].

The Volume-Viscosity Swallow Test (VVST; [17]) is a dysphagia screen that expands on water-swallowing tests by including 5- to 20-cc amounts of pudding and nectar-thick consistencies in addition to water, monitoring for overt signs of aspiration, but does not assess hard solids or masticatory function. The Mann Assessment of Swallowing Ability (MASA; [18]) includes assessments of oral preparation, bolus clearance, oral transit, and overt aspiration signs. However, this assessment yields global severity scores that are based on subjective judgements rather than objective measures, introducing the risk of bias. Similarly, the TOR-BSST [19] relies upon subjective judgements of voice, tongue movement, and water swallowing, but was designed as a screening tool, rather than to quantify the oral phase of swallowing.

The Iowa Oral Performance Instrument (IOPI) is a measurement tool which allows for the objective measurement of orofacial strength and endurance, from which physiologic reserve can be derived. While extensive normative data for measures such as maximum lingual strength are available (e.g. [20–22]),, data supporting the relationship between these measures and functional oral phase swallowing outcomes are limited.

The Test of Masticating and Swallowing Solids (ToMaSS) was consequently developed to quantify solid bolus ingestion as an accompanying quantitative assessment tool to the CSE [23]. Similar to methods for the TWST, this test requires a patient to eat a single cracker “as quickly as is comfortably possible, and when you have finished, say your name out loud” [24]. Four quantitative measures are recorded: the number of bites, masticatory cycles, swallows, and total time required for solid bolus ingestion. The number of swallows are identified by visual observation of thyroid cartilage movement; a stopwatch is used to record the time from the moment the cracker passes the bottom lip until the individual says their name; the number of bites are counted by the number of discrete segments of a cracker placed in the mouth and the number of masticatory cycles are counted through observation of superior-inferior jaw movement [24]. In this way, information regarding efficiency of the oral phase of swallowing is gained [25].

Recent studies have demonstrated that the ToMaSS has moderate to excellent intra-rater, inter-rater, and test-retest reliability in both healthy adults [11, 24, 26, 27] and patients with neurogenic dysphagia [23, 27–29]. Construct validity has been supported by strong correlations between ToMaSS measures and surface electromyography [24], and concurrent validity by agreement between ToMaSS and endoscopic outcomes for (number of) *Swallows* [27]. The ToMaSS has also shown sensitivity to oral-phase changes following topical anaesthetic application particularly the number of masticatory cycles and total ingestion time [25]. Results comparing different commercially available crackers on ToMaSS measures have been mixed: Huckabee et al. [24] found differences across all measures between two similar crackers (Arnott’s Salada™ and Nabisco Saltine™), whereas Hägglund et al. [30] found no significant differences between two cracker types available in Scandinavia, possibly reflecting differences in methodology and sample size.

Normative data have since been collected across a range of crackers found in different regions – including North America, Australasia, Europe and Asia [11, 24, 26, 30, 31]. These studies consistently report mean values and 95% confident intervals or standard deviations stratified by sex and age bands, though small group sizes (typically *n* = 15–20 per group) remain a significant limitation. For example, in the study by Huckabee et al. [24], although there was a total sample size of *N =* 124, this represented just *n =* 15–17 individuals per age/sex group. More recent work has adopted the digital ToMaSSApp™ [32, 33] – a digital application designed to improve the efficiency of acquiring ToMaSS data [33, 34].

Small intra-group sample sizes such as these challenge the representation of norms for individual age and sex groups, leaving norms at risk of skew, influence by outliers, and reduced statistical power, particularly in the older age groups where higher variability is expected [32, 33]. As a result, these normative data sets are unlikely to represent true means or even true variance that is representative of the population [32, 33]. A large sample size is needed to help mitigate these issues and provide consistent and reliable normative data that accurately represent the healthy adult population or an alternate method of data analysis is required.

Regression-based norming was first introduced by Zachary and Gorsuch [34] as a more efficient alternative to traditional norming. Traditional norming is straightforward, with norm statistics computed directly from the distribution of test scores within predefined subgroups. This simplicity enhances its clinical application, as no complex computations are required. However, the primary disadvantage of this method is the arbitrary division of continuous covariates, such as age, into mutually exclusive and exhaustive categories. This can lead to variations in interpreting an individual’s test performance depending on the assigned norm group [35]. While defining more categories might address some biases, it results in smaller sample sizes for each category, leading to normative data with lower precision.

In contrast, regression-based norming uses covariates such as age and sex as predictors in a regression model, allowing for more efficient use of the entire sample. By avoiding arbitrary subgroup divisions, this approach enhances the precision of test score estimates. Individuals at the far ends of the stratified group are no longer biased to be at the far ends of the normative sample if performance changes relative to age. A study by Oosterhuis et al. [36] demonstrated that under the assumptions of linear regression regression-based norming can reduce sample size requirements by 2.5 to 5.5 times compared to traditional methods, particularly when subdividing samples into groups.

While the assumptions of linear regression — such as normally distributed errors, homoscedasticity, and linearity — are important to consider and can impact the validity of the norms [37], the overall potential for regression-based norming to streamline the norming process and improve accuracy is significant. This method not only provides accurate estimates with fewer participants but also simplifies the complexity of large sample collections, making it particularly valuable in test construction and norm development. Nonlinear regression models may also be considered to mitigate strict assumptions, further enhancing the robustness of the approach [38–41].

## Aim

The aim of this study was to investigate the effectiveness of regression-based norming for the ToMaSS. Specifically, the study evaluated whether regression-based norms would provide more statistically accurate and precise estimates compared to published traditional norming methods.

## Methods

### Participants

This study used a prospective-retrospective design, where data were prospectively collected and combined with historical data from published [24, 42] and unpublished laboratory datasets [43–45]. Historical datasets were selected based on methodological similarities to the prospective data, including the same cracker type and the same metropolitan location. Data were collected by trained clinical researchers by manually counting and recording the primary outcome measures, with one exception: the study by Mills et al. [44] used the ToMaSSApp™. However, recent work comparing app-based and manual counts [46] demonstrated excellent absolute agreement for measures of Time, Bites, and Masticatory Cycles (ICC = 0.94–0.99) and good agreement for number of Swallows (ICC = 0.79) [47], with low mean bias identified across all variables, supporting ToMaSSApp™ as a valid substitute for manual counting and appropriate for inclusion in the present study. All data consisted of healthy adults (aged 20 to 80 + years), recruited from the general public. In this context, healthy was defined as no self-reported history of speech, language, neurological, or swallowing disorders, and the absence of dysphagia symptoms, active dental changes (e.g. infection, recent extraction, etc.) or major medical diagnoses affecting oromotor function. Participants were divided into groups according to age and sex per 20-year age group (i.e., ages 20–40, 41–60, 61–80, 80–100). A minimum of 50 males and 50 females were included per 20-year age group. Sample size calculation was based on a study by Bridges and Holler [32] which found that normative studies should aim to include at least 50 individuals per group. The study was approved by the appropriate university Human Research Ethics Committee (HEC 2021/106/LR Amendment 1).

### Materials

The Arnott’s Salada™ cracker was used in all studies and is readily available throughout New Zealand and Australia. Each wheat-based cracker weighs 3.75 g with dimensions of 5 cm^2^.

### Procedure

Procedures were identical for prospective and retrospective datasets, with the exception of Mills et al. [44] where the ToMaSSApp™ was used for data recording. Participants were instructed to eat the cracker ‘as quickly as is comfortably possible. Say your name when you are done’. Participants were observed from the side, and ToMaSS measures (*Bites*,* Masticatory cycles*,* Swallows*,* and Time in seconds*) were recorded either manually (pencil and paper) or using an app developed to facilitate data collection (ToMaSSApp™). Operational definitions for outcome variables were as follows: (1) *Bite* – the discrete segments taken into the mouth to ingest the entire cracker, (2) *Masticatory cycle* – an upwards and downwards movement of the jaw; lateral jaw movement was not counted, (3) *Swallow* – determined visually, by observed superior movement of the thyroid cartilage, (4) *Time* – the time in seconds from when the cracker first passed the bottom lip to when the participant said their name. These observational measures have been previously validated to instrumental measures and reliability established [24].

## Data analysis

All analyses were performed using R statistical environment [48].

### Regression Based Norms Model Selection

To calculate regression-based norms, various statistical models were employed based on the characteristics of each outcome measure. Linear Models (LM) and Generalised Linear Models (GLMs) with a Gamma family distribution were used for *Time* and *Masticatory cycles*, while Poisson Models (PM) were applied to *Bites* and *Swallows*. Generalised Additive Models (GAMs) were used when a non-linear relationship between age and the outcomes was detected, with the appropriate family distribution selected accordingly. Interactions between age and sex were included in all analyses.

For the regression approach to the data set, the model selection process began by verifying that necessary assumptions (e.g., normality, homoscedasticity, and non-dispersion) were satisfied. When multiple models met these assumptions, the Akaike Information Criterion (AIC) and Bayesian Information Criterion (BIC) were computed, and the model with the lowest AIC/BIC was chosen as the final model.

Traditional regression norms were established on the same data set by stratifying the data into four groups based on previously published age ranges (e.g., 20–40, 41–60, 61–80, 80+) for males and females separately [24]. Predicted means were calculated for the outcome variables across different ages and sexes for regression norms and across the stratified groups for traditional norms, along with 95% confidence intervals (CIs).

### Comparison of Regression-based Norms and Traditional Norms

To compare regression-based norms with traditional stratified norms, McFadden’s Pseudo R² and Root Mean Squared Error (RMSE) were calculated for both approaches. Pseudo R² is a widely used metric for GLMs and GAMs, quantifying the improvement in model fit relative to a null model using log-likelihood values. This provides insight into the model’s explanatory power in likelihood-based frameworks. To ensure a comparable pseudo R² for the traditional approach, this approach was treated as a Gaussian GLM, where predictions were based on group means. In contrast, RMSE measures the average difference between observed and predicted values, with lower values indicating better predictive accuracy.

Additionally, the impact of varying sample sizes on precision for both normative methods was examined. Equal numbers of observations were sampled from the eight age-sex groups of the traditional norms for specified sample sizes (80, 160, 240, 320, 400, 480, and 520), using sampling with replacement to ensure balanced representation. This process was repeated for 1,000 simulations for each sample size to capture variability in the estimates.

For the regression-based norms, confidence intervals (CIs) were calculated from the model’s predicted values and standard errors for each age. Since regression provides a unique CI for each age, the median CI width (the median difference between the upper and lower CI), and the median predicted value were computed across all ages within each age-sex group for comparison with traditional norms. For traditional norms, confidence intervals were obtained using a bootstrapping approach to calculate estimated means for each group, effectively addressing the lack of homogeneity of variance across groups.

The median 95% CI width and the standard deviation of the predicted value across the 1000 simulations for both methods for all ages and both sexes was calculated, highlighting how precision varies with sample size and methodological differences. The ratio of CI widths between the two methods was calculated by dividing the regression method’s CI width by that of the traditional method with values lower than 1 indicating greater precision.

### Regression-based Normative Data Calculation

To present the normative data from the final selected regression model, predicted means were calculated for each age and sex, along with 50%, 80%, 90%, and 95% prediction intervals. For the GAM models, these intervals were derived by generating new parameter vectors based on the estimated model parameters and simulating outcomes to account for both parameter uncertainty and natural data variability. Cholesky decomposition was applied to the covariance matrix when applicable, to preserve parameter correlations [49]. This approach enhances prediction reliability by considering both sources of uncertainty [50, 51]. For the Poisson models, prediction intervals were obtained by simulating 1,000 Poisson-distributed counts for each combination of predictor variables, based on the model’s predicted means.

## Results

### Participants

A total of 601 participants were included, with ages ranging from 20 to 99 years (Mean = 59.9 years) and 48% males. Of these, 453 participants (75%) were from the current study, while 148 (25%) represented historical data from previous studies. Table 1 presents the age groups and sex distribution of participants as defined by traditional norms.

**Table 1:** Distribution of participants by age group, including the number of females and males, total participants, and percentage of males across different age categories

Age Group (years)	Female (*n*)	Male (*n*)	Total (*n*)	Percentage of Males
20–40	68	68	136	50
41–60	75	60	135	44.4
61–80	94	105	199	52.8
80+	77	54	131	41.2
**Total**	**314**	**287**	**601**	**47.7**

### Regression Based Norms Model Selection

Due to observed strong non-linear relationships and increased variance of both *Time* and *Masticatory cycles* with age variables, a GAM with a Gamma distribution and a GLM with a Gamma distribution were the only models that satisfied the assumptions. The GAM Gamma model had slightly lower BIC and AIC values than the GLM Gamma model, making it the preferred choice (Table 2). This model accounted for the smooth effect of Age varying by sex and was estimated using REML.

For both Bites and Swallows, the Conway-Maxwell-Poisson (COM-Poisson) model was the only one that met the assumptions due to the data’s dispersion and low-count nature. Since zero values were absent (e.g., the number of bites or swallows cannot be zero) and a zero-truncated COM-Poisson model is not available in R, the outcome variables were adjusted by subtracting one from each observation to allow zeros in the distribution. This transformation did not affect model predictions, so one was added back to the reported means and confidence intervals to restore the original scale after the predicted values were calculated.

**Table 2:** Comparison of models using Akaike Information Criterion (AIC) and Bayesian Information Criterion (BIC) for the *Time* and *Masticatory cycles* outcome variables

Outcome Variable	Model	AIC	BIC
Time	GLM Gamma	4870.18	4892.18
	**GAM Gamma**	**4845.35**	**4890.85**
Masticatory cycles	GLM Gamma	5045.37	5067.35
	**GAM Gamma**	**5008.55**	**5057.30**

^a^GLM, generalised linear model

^b^GAM, generalised additive model

^c^AIC, Akaike Information Criterion

^d^BIC, Bayesian Information Criterion

^e^Lower AIC and BIC values reflect better model fit, with the final selected model highlighted in **bold**

### Comparison of Regression-based Norms and Traditional Norms

The Mean Squared Error and R-squared values were higher for all regression-based norms compared to the traditional norms, suggesting that the regression models provided better precision (Table 3). Regression-based norms (GAM Gamma and COM-Poisson models) showed a modest improvement over traditional stratified norms, with McFadden’s Pseudo R² increasing by 6% to 43% across outcomes. The greatest relative improvement was observed for *Time* (+ 42.6%) and *Masticatory cycles* (+ 36.9%), where GAM models demonstrated better model fit and predictive accuracy. For *Bites* (+ 7.5%) and *Swallows* (+ 7.4%), the differences were smaller, indicating similar performance between approaches. MSE values followed a similar trend, with regression-based norms reducing prediction error by up to 12% for *Time*.

**Table 3:** Model comparison for McFadden’s Pseudo R² and Mean Squared Error for *Time*,* Masticatory cycles*,* Bites*, and *Swallows* outcome variables

Outcome Variable	Model	McFadden’s Pseudo *R*²	MSE
Time	GAM Gamma	0.067	259.79
	Traditional norms	0.047	296.43
Masticatory cycles	GAM Gamma	0.063	13.19
	Traditional norms	0.046	13.86
Bites	COM-Poisson	0.115	1.49
	Traditional norms	0.107	1.52
Swallows	COM-Poisson	0.029	1.12
	Traditional norms	0.027	1.10

^a^GAM, generalised additive model

^b^COM-Poisson, Conway-Maxwell-Poisson

^c^MSE, Mean Squared Error

^d^Higher R-squared and lower MSE values indicate better model performance

**Fig. 1:**
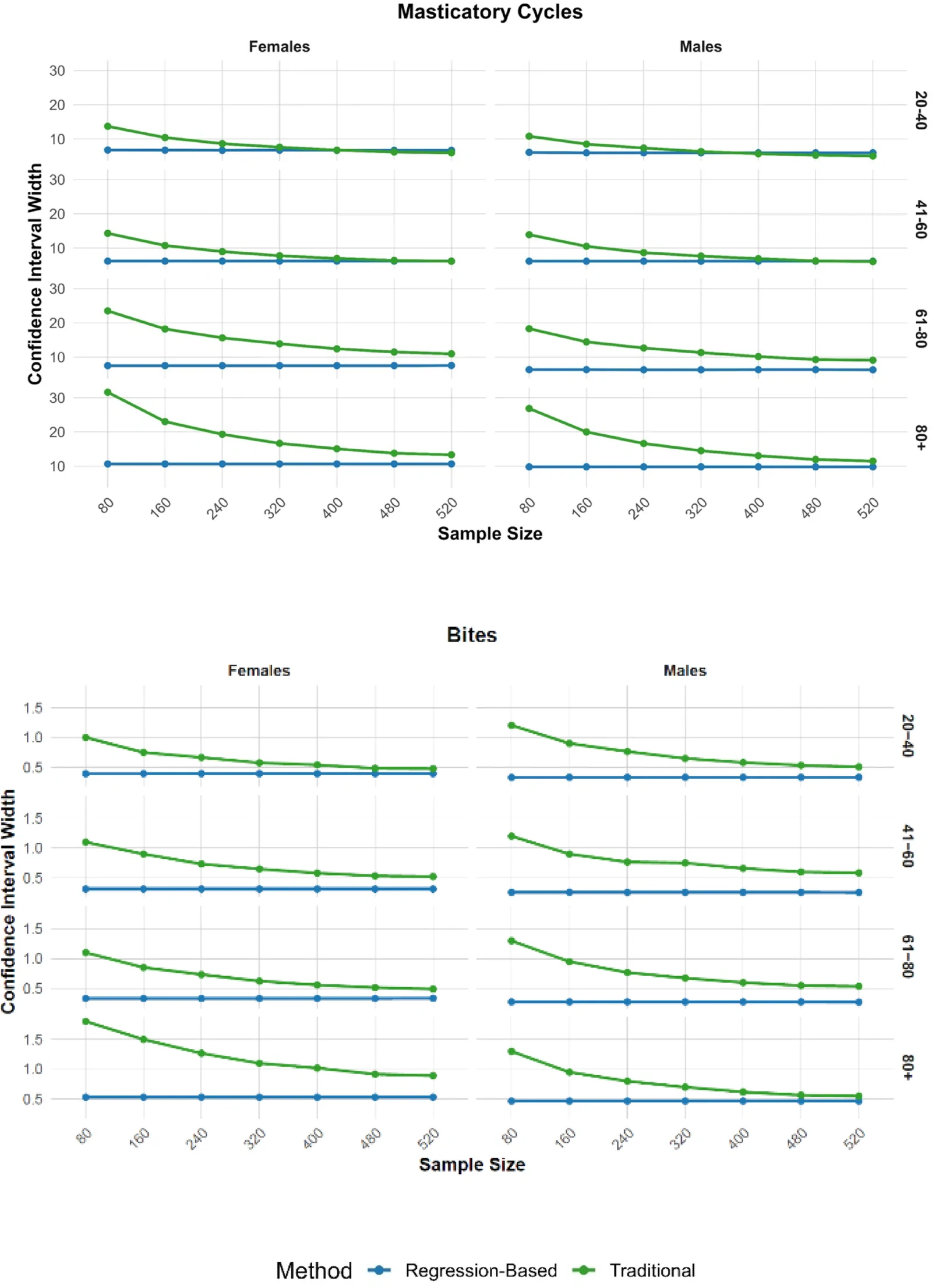

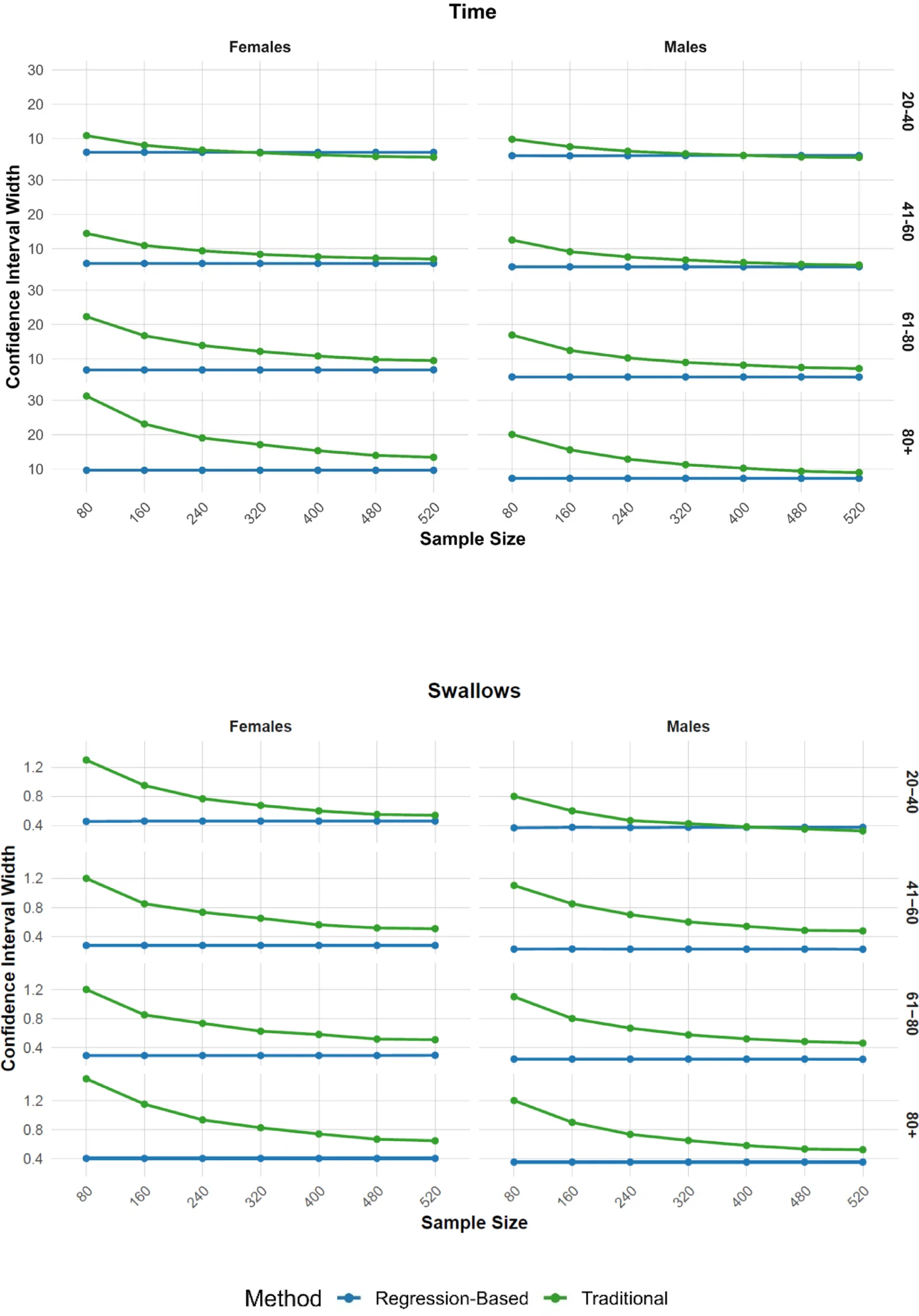
Comparison of regression-based and traditional methods for determining ToMaSS outcomes (*Masticatory cycles*,* Time*,* Bites*,* Swallows*). Confidence interval width by sample size, age group, and sex are displayed. Blue lines correspond to regression-based norms; green lines represent traditional norms

For each age group and sample size, regression-based norming demonstrated higher estimation precision, as indicated by a smaller median 95% CI width (Fig. 1). The difference between the two methods was largest for smaller sample sizes, females, and older age groups, gradually decreasing with increasing sample size. This difference levelled off at approximately *n* = 400 (50 per age group) for the 20–40 years and 41–60 years groups, while remaining slightly superior for the 61–80 years and 80 + years age groups.

The Bites outcome variable was the only case where males showed wider confidence intervals than females for the traditional norming approach, suggesting greater variability in this measure. For regression-based norms, CI widths remained stable across sample sizes, with only minimal reductions as sample size increased. In contrast, traditional norms showed a steep decline in CI width as sample sizes grew, indicating that they were more sensitive to sample size fluctuations.

Table 4 summarizes the average, minimum, and maximum of the confidence interval (CI) width for traditional and regression-based norming across all sample sizes. The 95% CI width ratio ranged from 0.21 to 1.18, with all average ratios below 1, indicating that regression-based norms generally provided narrower CIs than traditional norms. This was more pronounced in older age groups, which had smaller ratios compared to younger groups. The lowest average ratio was 0.39 for *Bites* in males aged 61–80 years, meaning that the CI width for traditional norming was 2.56 times larger than that of regression-based norming. The largest average ratio was 0.99 for *Time* in females aged 20–40 years, indicating nearly identical precision between the two methods.

**Table 4:** Average (minimum, maximum) confidence interval (CI) width ratios across all sample sizes evaluated in the analysis comparing traditional and regression-based norming methods across age groups and sex

		Confidence Interval Width Ratio (minimum, maximum)			
Age Group (years)	Sex	Masticatory cycles	Time	Bites	Swallows
20–40	F	0.86 (0.49, 1.12)	0.99 (0.55, 1.30)	0.65 (0.39, 0.83)	0.65 (0.35, 0.80)
	M	0.91 (0.56, 1.18)	0.87 (0.51, 1.12)	0.49 (0.27, 0.65)	0.85 (0.46, 1.10)
41–60	F	0.76 (0.43, 1.01)	0.65 (0.39, 0.81)	0.47 (0.29, 0.60)	0.42 (0.23, 0.54)
	M	0.78 (0.44, 1.02)	0.68 (0.38, 0.90)	0.35 (0.22, 0.44)	0.36 (0.21, 0.40)
61–80	F	0.53 (0.32, 0.69)	0.54 (0.30, 0.72)	0.52 (0.30, 0.69)	0.44 (0.24, 0.58)
	M	0.55 (0.35, 0.69)	0.50 (0.28, 0.66)	0.39 (0.21, 0.51)	0.40 (0.22, 0.52)
80+	F	0.61 (0.34, 0.80)	0.55 (0.31, 0.72)	0.47 (0.30, 0.60)	0.47 (0.27, 0.62)
	M	0.65 (0.34, 0.85)	0.62 (0.36, 0.81)	0.65 (0.36, 0.84)	0.52 (0.29, 0.67)

^a^F, Female

^b^M, Male

^c^Ratios less than 1 indicate narrower confidence intervals for regression-based norming, suggesting more precise estimates

**Fig. 2:**
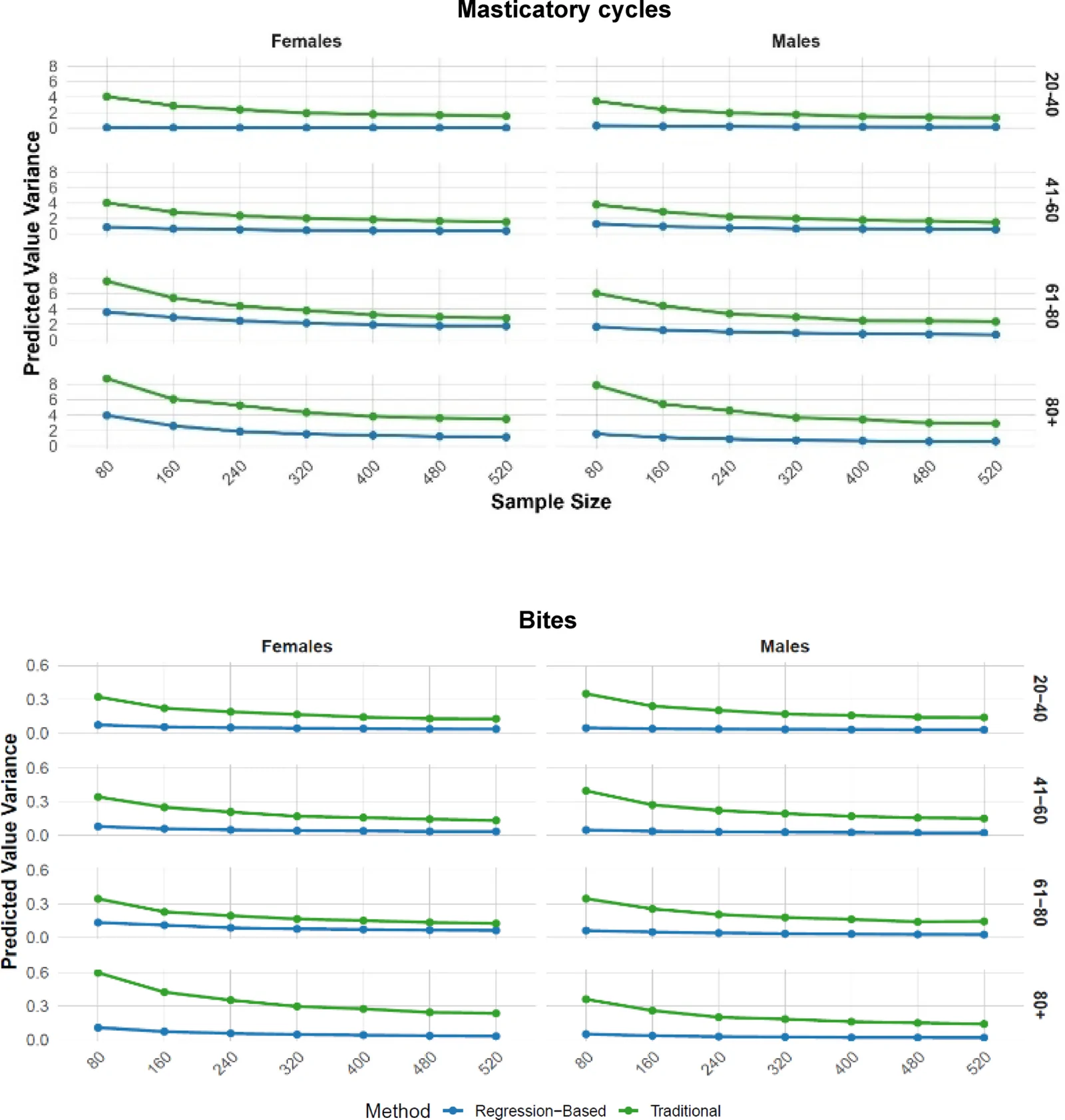

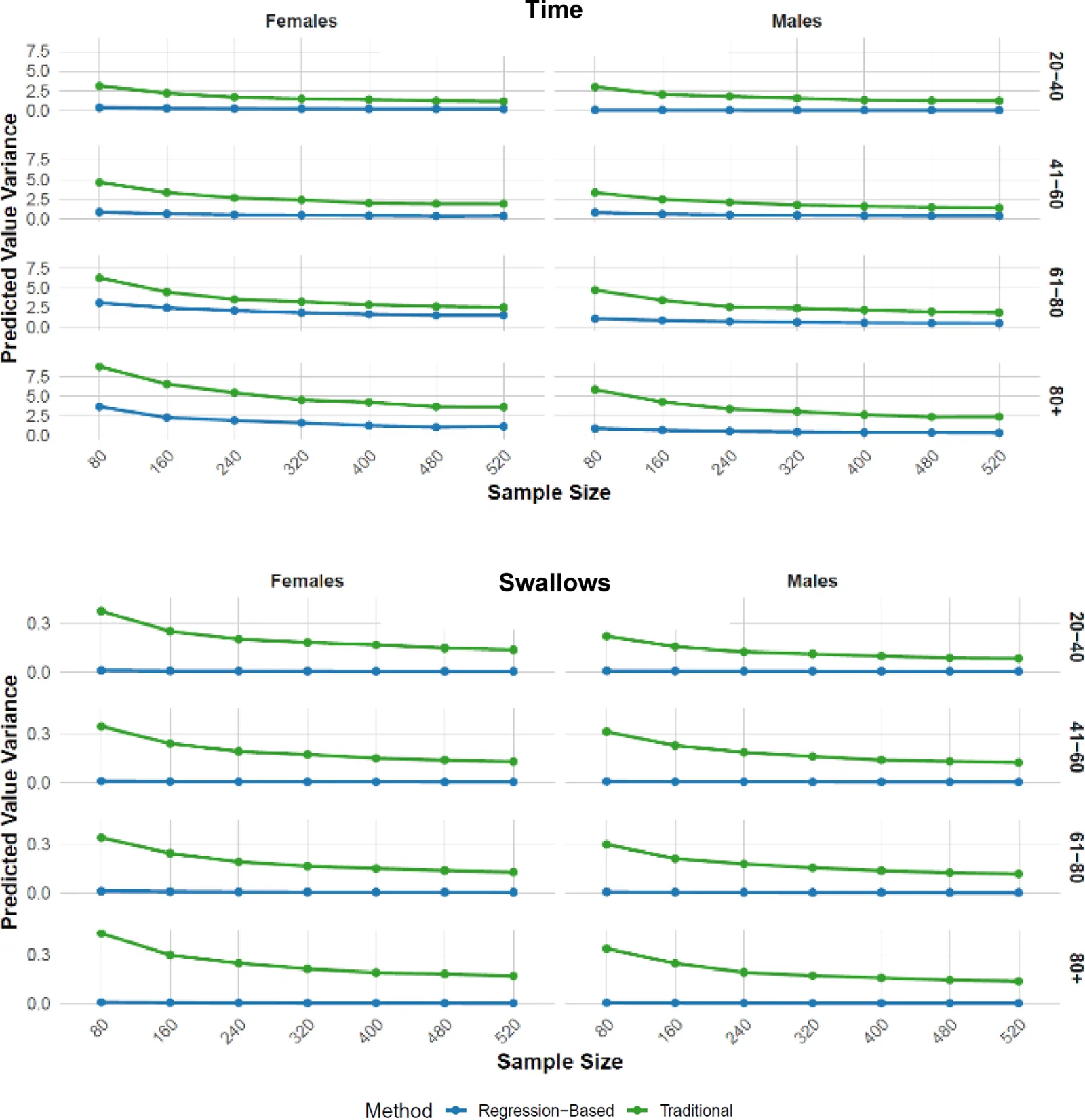
Comparison of estimated value variability for all ToMaSS outcome variables (*Masticatory cycles*,* Bites*,* Time*,* Swallows*) between regression-based norming (blue) and traditional norming (green) across age groups and sexes, with varying sample sizes. Precision is represented by the standard deviation of estimated values across 1,000 simulations. Predicted value variance is measured in the units of the respective ToMaSS outcome variable

The variability of predicted values, measured as the standard deviation of estimated outcome measures across 1,000 simulations, was consistently higher for traditional norms than for regression-based norms across all age groups, sexes, and sample sizes (Fig. 2). The difference was most pronounced for smaller sample sizes, where traditional norms exhibited greater fluctuations. As sample size increased, variability decreased for traditional norming, whereas regression-based norms remained more stable and consistently lower, with only minimal reductions as sample size increased.

### Regression-based Normative Data Calculation

**Fig. 3:**
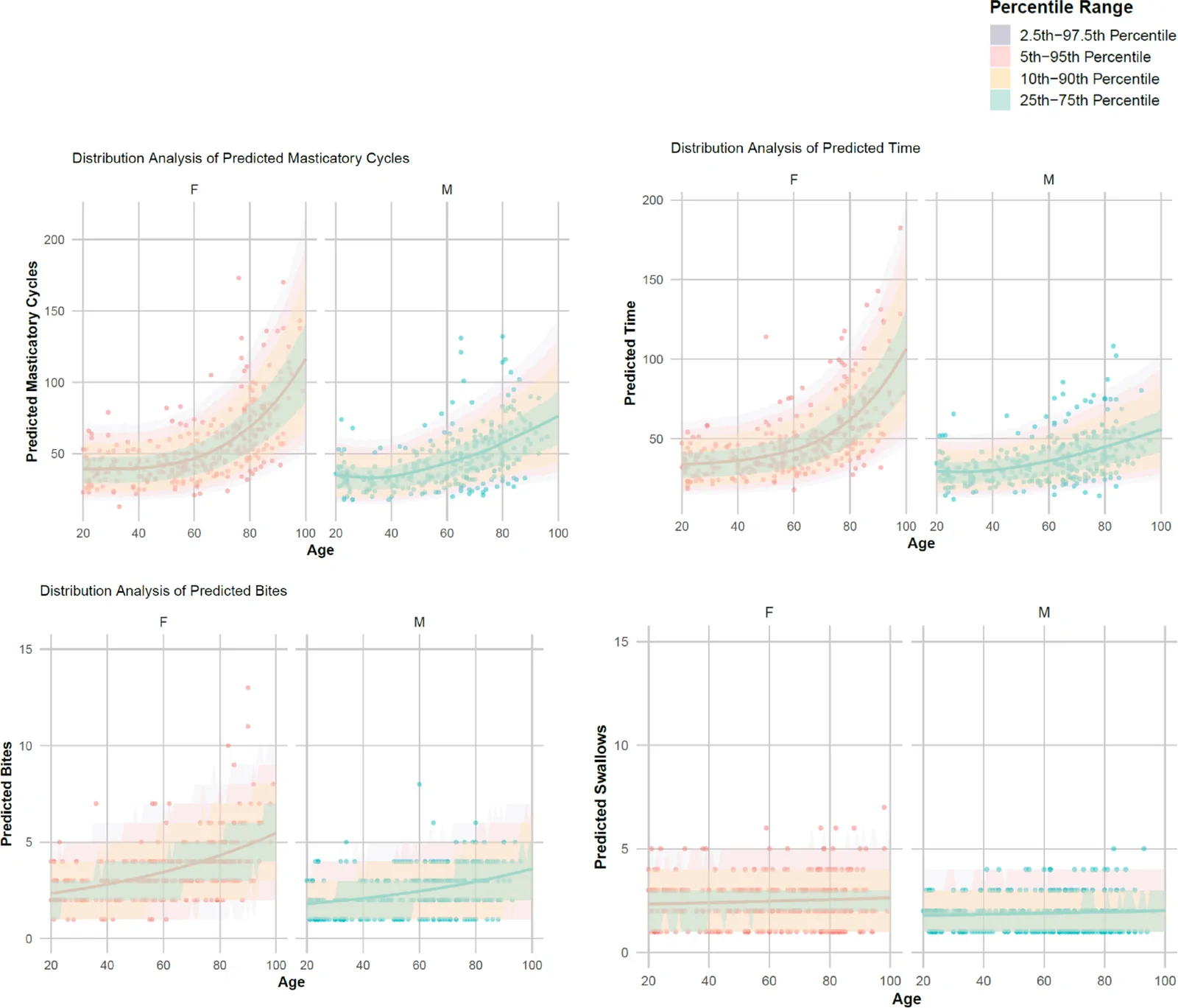
Predicted percentile values for *Masticatory cycles*,* Time*,* Bites*,* and Swallows* by age and sex. The data points represent individual predictions, while the shaded regions indicate percentile ranges (2.5th–97.5th, 5th–95th, 10th–90th, and 25th–75th percentiles) to capture variability

The number of predicted *Masticatory cycles* showed an increasing trend with age, with a more pronounced rise in female older individuals. A similar trend was observed in predicted *Time*, where the duration of the test increased with age, particularly in older females, who exhibit greater variability compared to males. In contrast, predicted *Bites* remained relatively stable across ages, with a slight increase in older individuals. Similarly, predicted *Swallows* showed a relatively stable distribution across age, with little change in males and females. Figure 3 presents the distribution of predicted percentiles for various ToMaSS metrics, stratified by age and sex.

## Discussion

This study demonstrated that a regression-based approach offers significant advantages over traditional stratified methods for refining ToMaSS normative data, potentially enhancing the clinical interpretation of ToMaSS scores. Previous normative studies using the ToMaSS provide valuable, baseline data across different crackers and demographics, but have consistently relied on age-sex stratification with relatively small numbers per age and sex group. Stratified norming such as this assigns identical reference values to all individuals within arbitrary, broad age and sex groups, leading to loss of granularity. This can be particularly problematic when a patient’s characteristics fall near a boundary between groups. In the present study, we compared a regression-based approach to the traditional stratified approach, and found that the regression-based approach achieved better precision – as indicated by higher R-squared values, narrower confidence intervals, and reduced MSE – compared to traditional norms.

Regression-based norms generate a continuous function of expected performance based on age and sex, enabling clinicians to interpret a patient’s performance in the context of their specific demographic profile. This affords several clinical benefits. First, it may increase diagnostic precision, reducing the risk that normal age-related variability is misclassified as impairment. Second, it enables more sensitive tracking of change over time, as small but clinically meaningful deviations from a patient’s predicted performance can be detected year-by-year. Third, regression-based norms support personalised assessment, providing a clear estimate of how far a patient’s performance deviates from someone with the same demographic characteristics. These advantages remain, for now, inferential – based on improved model precision and individualisation – and have not yet been validated in patient populations. However, collectively, these advantages have the potential to enhance the clinical utility of the ToMaSS by facilitating a more nuanced differentiation between normal variability and pathological change, supporting evidence-based decision-making in dysphagia assessment and management.

In addition, regression models provide accurate normative estimates and stable confidence intervals in the context of relatively smaller sample sizes. This contrasts with traditional stratified norms, which typically require large cohorts within predefined age groups to obtain consistent and reliable estimates. These key features should enable the development of robust ToMaSS normative data more efficiently, enabling broader clinical implementation across diverse populations and clinical settings. Findings highlighted that regression-based norms improved model performance, particularly for *Time* (+ 42.6%) and *Masticatory cycles* (+ 36.9%), where GAM models effectively accounted for non-linear age-related changes. To place the results in context, these models suggest that aging likely influences the mechanical effort required for mastication (which may be related to reduced muscle endurance) to a greater extent than for other variables, such as biting and the number of swallows, which are discrete acts. While the differences in model fit for *Bites* (+ 7.5% improvement) and *Swallows* (+ 7.4% improvement) were smaller, regression-based norms provided the advantage of generating specific percentiles for any given age rather than relying on broad, 20-year age categories.

There are further implications of using regression-based ToMaSS norms for both clinical research and clinical practice. The ToMaSS itself is a relatively inexpensive tool, requiring only a cracker and timer to administer. A regression-based norms approach reduces the need for extensive participant recruitment and leverages existing data more effectively, thus improving the feasibility of using this test in low-resource settings where large cohort studies are challenging to implement. Clinically, the flexibility of this approach allows for a more nuanced understanding of individual ToMaSS performance, and repeated testing over time. This may be particularly relevant for paediatric, geriatric, and neurologic populations, where age- and disease-related changes in swallowing may vary greatly within a short time frame.

### Limitations

Despite the advantages of regression-based norms, this study also highlights some limitations. First, while the models demonstrated improved predictive accuracy compared to traditional norms, a considerable portion of variability in outcomes remained unexplained. This suggests that additional variables – for example, dentition, frailty, and medication use —could be incorporated to refine estimates and enhance precision. The ability to include additional explanatory variables/control for confounders is a relative strength of the regression-based approach. Future research should explore the inclusion of such factors to further optimize normative predictions.

Second, implementing regression-based norms requires expertise in statistical modelling, which may limit accessibility for clinical centres without dedicated statistical support. Unlike traditional stratified norms, which can be easily applied using simple lookup tables, regression models necessitate specialized knowledge and software for data analysis and interpretation. As a result, healthcare facilities may need to rely on statisticians or specialized personnel to establish and maintain their normative datasets. A simple app or data calculator could be easily developed and distributed.

Third, the use of pooled, historical data can introduce the risk of population-specific variability and bias. In this case, all datasets were collected within the same laboratory using a consistent procedure, materials, and participant recruitment strategy. As such, methodological consistency and participant homogeneity was largely maintained and we are confident that historical data represent a single, well-controlled population sample. It is worth noting that, in this instance, the use of a well-controlled sample is a relative strength. However, future efforts in this area should take care to collect data from a broad and generalisable sample. Given that different regions have different availability of crackers, and that ToMaSS outcomes differ significantly between different cracker types [24], the authors recommend that independent regression-based norms be generated for each cracker type.

Finally, it is important to note that the models used in the present study were developed for normative prediction (i.e., estimating a continuous mean value), as opposed to probabilistic prediction or risk classification. As such, a separate calibration stage was not required, as calibration was inherently addressed through standard regression diagnostics, namely, inspection of residuals, assessment of model assumptions, and evaluation of model fit statistics (e.g., MSE, AIC/BIC). These checks confirmed that the models did not systematically over- or under-predict ToMaSS outcomes across age or sex. Future work could evaluate external validity in new samples, which would provide an opportunity to formally assess calibration across populations.

## Conclusion

Regression-based norms for the ToMaSS achieved better precision – as indicated by higher R-squared values, narrower confidence intervals, and reduced MSE – compared to traditional norms. The regression-based approach is specific and personalisable – allowing for the assessment of patient performance in the context of age and sex, as compared to interpretation within broad age categories. Overall, these findings reinforce that regression-based norms provide a more efficient and scalable approach to establishing normative data for the ToMaSS. By requiring fewer participants while maintaining precision, this method supports the expansion of ToMaSS normative datasets across different settings, ultimately improving clinical uptake of this assessment and its applicability in diverse patient cohorts.

## References

[1] McCullough GH, Wertz RT, Rosenbek JC, Dinneen C. Clinicians’ preferences and practices in conducting clinical/bedside and videofluoroscopic swallowing examinations in an adult, neurogenic population. Am J Speech Lang Pathol. 1999;8(2):149–63. 10.1044/1058-0360.0802.149.

[2] McCullough GH, Rosenbek JC, Wertz RT, McCoy S, Mann G, McCullough K. Utility of clinical swallowing examination measures for detecting aspiration post-stroke. J Speech Lang Hear Res. 2005;48(6):1280–93. 10.1044/1092-4388(2005/089). 16478371

[3] Mathers–Schmidt BA, Kurlinski M. Dysphagia evaluation practices: inconsistencies in clinical assessment and instrumental examination decision-making. Dysphagia. 2003;18(2):114–25. 10.1007/s00455-002-0094-z. 12825905

[4] Martino R, Pron G, Diamant NE. Oropharyngeal dysphagia: surveying practice patterns of the speech-language pathologist. Dysphagia. 2004;19(3):165–76. 10.1007/s00455-004-0004-7. 15383946

[5] Vogels B, Cartwright J, Cocks N. The bedside assessment practices of speech-language pathologists in adult dysphagia. Int J Speech Lang Pathol. 2015;17(4):390–400. 10.3109/17549507.2014.979877. 25495261

[6] McCullough GH, Wertz RT, Rosenbek JC, Mills RH, Ross KB, Ashford JR. Inter- and intrajudge reliability of a clinical examination of swallowing in adults. Dysphagia. 2000;15(2):58–67. 10.1007/s004550010002. 10758187

[7] Nathadwarawala KM, Nicklin J, Wiles CM. A timed test of swallowing capacity for neurological patients. J Neurol Neurosurg Psychiatry. 1992;55(9):822–5. 10.1136/jnnp.55.9.822. PMC1015108 1402974

[8] Hughes TAT, Wiles CM. Clinical measurement of swallowing in health and in neurogenic dysphagia. QJM: An International Journal of Medicine. 1996;89(2):109–116. 10.1093/qjmed/89.2.109 8729551

[9] Sarve A, Krishnamurthy R, Balasubramanium R. The timed water test of swallowing: reliability, validity, and normative data from Indian population. Int J Health Sci (Qassim). 2021;15(2):14–20. PMC7934130 33708040

[10] Hägglund P, Karlsson P, Karlsson F. The timed water swallow test (TWST): normative data on swallowing capacity for healthy people aged 60 years and older. Int J Speech Lang Pathol. 2023;25(4):479–85. 10.1080/17549507.2022.2096925. 35819269

[11] Sella-Weiss O. The test of mastication and swallowing solids and the timed water swallow test: reliability, associations, age and gender effects, and normative data. Int J Lang Commun Disord. 2023;58(1):67–81. 10.1111/1460-6984.12768. PMC10087873 36036753

[12] Martell P, Skogar Ö, Bergström L. Swallowing characteristics and water swallow capacity in patients with Parkinsonism. Dysphagia. 2024. 10.1007/s00455-024-10685-3. PMC11606992 38703231

[13] Watson L, Woodman SH, Ganderton D, Hutcheson KA, Pringle S, Patterson JM. Development of the remote 100 ml water swallow test versus clinical assessment in patients with head and neck cancer: do they agree? Head Neck. 2022;44(12):2769–78. 10.1002/hed.27194. 36114683

[14] Vasconcellos P, Mafort TT, Ribeiro-Alves M, da Costa CH. Association between swallowing dynamics, tongue pressure and pulmonary function in patients with idiopathic pulmonary fibrosis. BMC Pulm Med. 2024. 10.1186/s12890-024-03117-3. PMC11223328 38965496

[15] Sayaca C, Sapcióglu TY, Kaya D. Does the right or left hemisphere involvement affect swallowing in patients with hemiplegia? NeuroRehabil. 2020;46(4):501–9. 10.3233/NRE-203089. 32538883

[16] Reyes A, Cruickshank T, Nosaka K, Ziman M. Respiratory muscle training on pulmonary and swallowing function in patients with Huntingtons disease: a pilot randomised controlled trial. Clin Rehabil. 2015;29(10):961–73. 10.1177/0269215514564087. 25552526

[17] Clavé P, Arreola V, Romea M, Medina L, Palomera E, Serra-Prat M. Accuracy of the volume-viscosity swallow test for clinical screening of oropharyngeal dysphagia and aspiration. Clin Nutr. 2008;27(6):806–15. 10.1016/j.clnu.2008.06.011. 18789561

[18] Mann G. MASA: The Mann Assessment of Swallowing Ability. Clifton Park, NY: Thomson Delmar Learning; 2002.

[19] Martino R, et al. The Toronto Bedside Swallowing Screening Test (TOR-BSST): development and validation of a dysphagia screening tool for patients with stroke. Stroke. 2009;40(2):555–61. 10.1161/STROKEAHA.107.510370. 19074483

[20] Robbins J, Levine R, Wood J, Roecker EB, Luschei E. Age effects on lingual pressure generation as a risk factor for dysphagia. J Gerontol. 1995;50(5):257–62. 10.1093/gerona/50a.5.m257 7671027

[21] Clark HM, Solomon NP. Age and sex differences in orofacial strength. Dysphagia. 2012;27(2):2–9. 10.1007/s00455-011-9328-2. 21350818

[22] Youmans S, Youmans G, Stierwalt J. Differences in tongue strength across age and gender: is there a diminished strength reserve? Dysphagia. 2009;24:57–65. 10.1007/s00455-008-9171-2 18690406

[23] Athukorala RP, Jones RD, Sella O, Huckabee M-L. Skill training for swallowing rehabilitation in patients with Parkinson’s disease. Arch Phys Med Rehabil. 2014;95(7):1374–82. 10.1016/j.apmr.2014.03.001. 24816250

[24] Huckabee M-L, et al. The Test of Masticating and Swallowing Solids (TOMASS): reliability, validity and international normative data. Int J Lang Commun Disord. 2018;53(1):144–56. 10.1111/1460-6984.12332. 28677236

[25] Lamvik-Gozdzikowska K, Guiu Hernandez E, Apperley O, McIntosh T, Huckabee M. Quantitative assessment of oral phase efficiency: validation of the Test of Masticating and Swallowing Solids (TOMASS). Int J Lang Commun Disord. 2019;54(3):444–50. 10.1111/1460-6984.12450. 30663183

[26] Kothari S, Krishnamurthy R, Balasubramanium RK, Huckabee M-L. The Test of Masticating and Swallowing Solids (TOMASS): reliability, validity and normative data for the adult Indian population. Indian J Otolaryngol Head Neck Surg. 2021;73(3):327–32. 10.1007/s12070-021-02429-8. PMC8364578 34471621

[27] Todaro F, Pizzorni N, Scarponi L, Ronzoni C, Huckabee ML, Schindler A. The Test of Masticating and Swallowing Solids (TOMASS): reliability and validity in patients with dysphagia. Int J Lang Commun Disord. 2021;56(3):558–66. 10.1111/1460-6984.12613. 33687133

[28] Borders JC, Sevitz JS, Malandraki JB, Malandraki GA, Troche MS. Objective and subjective clinical swallowing outcomes via telehealth: reliability in outpatient clinical practice. Am J Speech Lang Pathol. 2021;30(2):598–608. 10.1044/2020_AJSLP-20-00234. 33555954

[29] Karlsson F, Lovric L, Matthelié J, Brage L, Hägglund P. A within-subject comparison of face-to-face and telemedicine screening using the Timed Water Swallow Test (TWST) and the Test of Mastication and Swallowing of Solids (TOMASS). Dysphagia. 2023;38(1):483–90. 10.1007/s00455-022-10490-w. PMC9873209 35809097

[30] Hägglund P, Blom S, Thoden P, Karlsson F. The Test of Masticating and Swallowing Solids (TOMASS): normative data for two crackers available in the Scandinavian and international markets. Int J Speech Lang Pathol. 2021;23(3):329–37. 10.1080/17549507.2020.1800090. 32867525

[31] Kavakci M, Tanrıverdí M, Barmak E, Kapan N. Turkish normative data for the Test of Masticating and Swallowing Solids (TOMASS). Cukurova Med J. 2021;46(3):920–6. 10.17826/cumj.840796.

[32] Bridges AJ, Holler KA. How many is enough? Determining optimal sample sizes for normative studies in pediatric neuropsychology. Child Neuropsychol. 2007;13(6):528–38. 10.1080/09297040701233875. 17852130

[33] Biau DJ, Kernéis S, Porcher R. Statistics in brief: the importance of sample size in the planning and interpretation of medical research. Clin Orthop Relat Res. 2008;466(9):2282–8. 10.1007/s11999-008-0346-9. PMC2493004 18566874

[34] Zachary RA, Gorsuch RL. Continuous norming: implications for the WAIS-R. J Clin Psychol. 1985;41(1):86–94. https://doi.org/10.1002/1097-4679(198501)41:1<86::AID-JCLP2270410115>3.0.CO;2-W. 10.1002/1097-4679(198501)41:1<86::aid-jclp2270410115>3.0.co;2-w 3973045

[35] Parmenter B, Testa S, Schretlen D, Weinstock-Guttman B, Benedict R. The utility of regression-based norms in interpreting the minimal assessment of cognitive function in multiple sclerosis (MACFIMS). J Int Neuropsychol Soc. 2010;16(1):6–16. 10.1017/S1355617709990750. 19796441

[36] Oosterhuis H, van der Ark LA, Sijtsma K. Sample size requirements for traditional and regression-based norms. Assessment. 2016;23(2):191–202. 10.1177/1073191115580638 25940350

[37] der Van Elst W, Hoogenhout E, Dixon R, De Groot R, Jolles J. The Dutch Memory Compensation Questionnaire: psychometric properties and regression-based norms. Assessment. 2011;18:517–28. 10.1177/1073191110370116 PMC3771363 20519736

[38] Semel E, Wiig EH, Secord WA. Clinical evaluation of language fundamentals — Screening test (CELF-4 screening test). 4th ed. Toronto: The Psychological Corporation/A Harcourt Assessment Company.; 2004.

[39] Tellegen P, Laros JA. Snijders-Oomen non-verbal intelligence test: Part I Motivation. Amsterdam: Hogrefe; 2011.

[40] Dinga R, Fraza CJ, Bayer JMM, Kia SM, Beckmann CF, Marquand AF. Normative modeling of neuroimaging data using generalized additive models of location scale and shape. Jun. 2021;14. 10.1101/2021.06.14.448106.

[41] Pinilla J, Negrín M. Non-parametric generalized additive models as a tool for evaluating policy interventions. Mathematics. 2021;9(4):299. 10.3390/math9040299.

[42] Ng KB, Jones RD, Hernandez EG, Macrae P, Huckabee M-L. Classification of stroke patients with dysphagia into subgroups based on patterns of submental muscle strength and skill impairment. Arch Phys Med Rehabil. 2021;102(5):895–904. 10.1016/j.apmr.2020.11.014. 33347889

[43] Lewis N. The Test of Masticating and Swallowing Solids (TOMASS) and the Timed Water Swallow Test (TWST): Adult Normative Data for the ToMaSSApp, Unpublished Master’s thesis, University of Canterbury, Christchurch, 2024.

[44] Mills M. The Test of Masticating and Swallowing Solids Application (ToMaSSAppTM): Normative data for two crackers, Unpublished Masters Thesis, University of Canterbury, Christchurch, 2024.

[45] Henderson A. Test of Masticating and Swallowing Solids: Sensitivity to Parkinson’s disease severity., Master’s thesis, University of Canterbury, Christchurch, 2015.

[46] Lewis N. The Test of Masticating and Swallowing Solids (ToMaSS) and the Timed-Water Swallow Test (TWST): Adult Normative Data for the ToMaSSApp (2024). [Master's dissertation, University of Canterbury].

[47] Koo TK, Li MY. A guideline of selecting and reporting intraclass correlation coefficients for reliability research. J Chiropr Med. 2016;15(2):155–63. 10.1016/j.jcm.2016.02.012. PMC4913118 27330520

[48] Team RC. R: A language and environment for statistical computing, R Foundation for Statistical Computing. [Online]. Available: https://www.r-project.org/

[49] Golub G, Van Loan C. Matrix Computations. 3rd ed. Johns Hopkins University; 1996.

[50] McCullagh P, Nelder J. Generalized Linear Models. 2nd ed. Chapman and Hall/CRC; 1989.

[51] Casella G, Berger R. *Statistical Inference*, 2nd ed. Duxbury, 2002.

